# Acrokeratosis paraneoplastica in serous ovarian carcinoma: case report

**DOI:** 10.1186/s12885-015-1527-z

**Published:** 2015-07-08

**Authors:** Aline Hempen, Eleftherios P Samartzis, Jivko Kamarachev, Daniel Fink, Konstantin J Dedes

**Affiliations:** 1 Department of Gynaecology, University Hospital Zurich, Frauenklinikstrasse, 8091 Zurich, Switzerland; 2 Department of Dermatology, University Hospital Zurich, Gloriastrasse 31, 8091 Zurich, Switzerland

**Keywords:** Paraneoplastic acrokeratosis, Bazex, Palmoplantar hyperkeratosis, Paraneoplasia, Serous ovarian cancer

## Abstract

**Background:**

Acrokeratosis paraneoplastica is a rare paraneoplastic phenomenon associated with upper aerodigestive tract carcinomas, usually manifesting as psoriasiform keratosis over the acral sites. It is primarily seen in white males above the age of 40 years. Here we report a case of paraneoplastic acrokeratosis in a woman with serous ovarian cancer. To the best of our knowledge, no similar case has been reported previously.

**Case presentation:**

We report the case of a 60-year-old woman diagnosed with a serous ovarian cancer and complaining of a thickening and peeling of the skin on her feet. Clinical and histological examination, as well as the course of disease, confirmed the diagnosis of a paraneoplastic plantar keratosis. Under systemic chemotherapy with carboplatin and paclitaxel the lesion resolved gradually in concordance with tumour marker CA 125.

**Conclusions:**

We present the reported case of paraneoplastic acrokeratosis associated with advanced high-grade ovarian cancer.

## Background

Acrokeratosis paraneoplastica of Bazex was first described by Bazex et al. in 1965 [1]. It is a rare acral hyperkeratosis, most commonly associated with squamous cell carcinoma of the upper aerodigestive tract or the cervicomediastinal area [2]. It can precede the diagnosis of the associated tumour up to 1 year. In most cases, it responds to successful treatment of the underlying tumour and fails to resolve when the neoplasm persists [3, 4]. In 18 % of patients, cutaneous lesions and associated malignancy are diagnosed concomitantly and in 15 % of the cases the diagnosis of cancer occurs first. In the remaining 67 % the cutaneous lesions precede the diagnosis of cancer [5]. The most common sites of involvement are the ears (79 %), nails (75 %), nose (63 %), hands (57 %) and feet (50 %). The histopathological findings are variable and include commonly acanthosis, psoriasiform epidermal hyperplasia, hyperkeratosis and parakeratosis. Sometimes perivascular lymphocytic infiltrate and dyskeratotic keratinocytes as well as vacuolar degeneration in the basal epidermal layer can be observed [6].

Paraneoplastic acrokeratosis affects primarily Caucasian men above 40 years of age. A review of 140 cases described only 12 cases occuring in women [4, 7].

We present the case of a 60-year-old woman with a newly diagnosed high-grade serous ovarian cancer and associated plantar paraneoplastic acrokeratosis. To our knowledge, the association of this carcinoma with Acrokeratosis paraneoplastica of Bazex has not been reported previously.

## Case presentation

A 60-year-old woman presented with acute upper abdominal pain, fever, nausea and shivering as well as elevated liver and pancreatic enzymes. A computed tomographic scan confirmed the suspicion of lithogenic pancreatitis but also revealed an unclear mass on both ovaries as well as signs of peritoneal carcinomatosis, omental cake, infiltration of the liver and spleen and substantial ascites at the same time.

CA125 tumour marker was elevated up to 305 U/mL suggesting the presence of an advanced ovarian cancer (normal value 0–35 U/mL). No other blood abnormalities beyond high tumour marker levels were found. Consequently a surgical tumour debulking was performed. Intraoperatively, a highly distinct peritoneal carcinomatosis with cementation of all the internal abdominal organs could be seen. The ovaries were buried under this tumourous mass and couldn’t be fully visualised during the operation. Under these circumstances no optimal cytoreduction could be performed. The histology later confirmed the presence of a high-grade serous carcinoma of the ovary. Chemotherapy with carboplatin and paclitaxel was induced postoperatively.

**Fig. 1:**
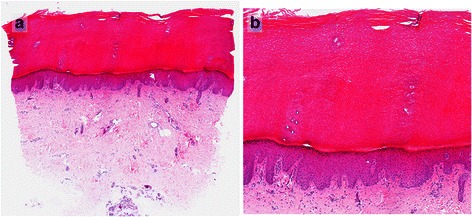
Biopsy was taken from the sole of the left foot: Histology of plantar skin showing compact hyperkeratosis and irregular epidermal hyperplasia without any elements of mycosis; enlarged 10 (**a**) and 40 (**b**) times

**Fig. 2:**
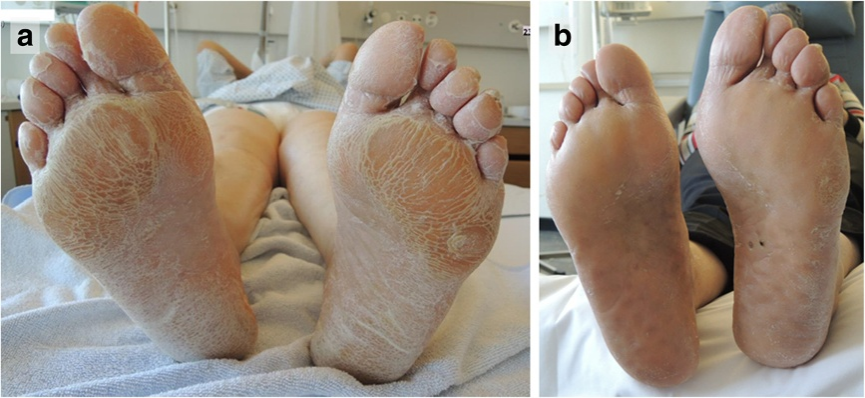
Laminar plantar hyperkeratosis on both feet with enhancement of areas under compressive stress (**a**) before treatment and (**b**) after three cycles of chemotherapy (punch marks on left sole)

Already prior to surgery, approximately when the ovarian cancer was diagnosed, the patient complained about a disturbing skin alteration on both her feet. Inspection revealed laminar hyperkeratosis and scaling of the plantar skin (enhanced in areas under compressive stress), which was unbeknownst to her. Dermatological examination as well as punch biopsy of the skin (Fig. 1a,b) suggested the presence of a paraneoplastic acrokeratosis. Histology of plantar skin revealed compact hyperkeratosis and irregular epidermal hyperplasia. Mycosis was excluded histologically. A regression of the cutaneous lesion together with the normalisation of the CA125 tumour marker (a drop to 9.3 U/mL) under first-line chemotherapy containing carboplatin and paclitaxel was observed, which further supported the suspected diagnosis of a paraneoplastic phenomenon (Fig. 2a,b). Cutaneous eruptions in other areas (eg. face, hands), as seen in some cases of paraneoplastic acrokeratosis, could not be found [4, 7].

## Discussion

Acrokeratosis paraneoplastica is a rare, obligate paraneoplasia initially presenting with palmoplantar hyperkeratosis. The pathogenesis is still not well understood. A few authors have proposed molecular mimicry as a possible mechanism; the crossed reactions between tumour antigens and growth factor receptors on epidermal cells. Another postulated mechanism is the action of epidermal growth factor, transforming growth factor-alpha and insulin-like growth factor secreted by the tumour cells themselves, which can lead to the cutaneous changes. Furthermore, zinc deficiency associated with the neoplasm may play a role in the pathogenesis of this hyperproliferative dermatosis. [2, 8, 9]. Hara et al. formulated a hypothesis where the pathomechanism of Acrotkeratosis paraneoplastica may be related to an immunological reaction as indicated by multiple factors: association with tumours always of epidermal and never of mesodermal origin, possible histopathological changes including the presence of necrotic foci, scattered vacuolar degeneration of basal cells, cytoplasmic eosinophilia and vacuolization of keratinocytes and pigmentary incontinence, which indicate an immunemediated attack on basal cells. The finding of immunoglobulin and C3 deposits on the basement membrane suggests the possibility of an antibody-initiated immune reaction, possibly mediated by complement, which provokes an inflammatory response resulting in development of the stated lesions [10].

Most cases of paraneoplastic acrokeratosis are associated with squamous cell carcinomas of the upper one-third of the respiratory or gastrointestinal tract (i.e. oral cavity, larynx, lungs, oesophagus). Single case reports also described an association of acrokeratosis paraneoplastica with carcinomas of the thymus, bladder, skin, multiple myeloma, carcinoids, malignant lymphomas and cholangiocarcinomas. Women seem to be less affected by acrokeratosis paranoplastica and only single cases have been reported in association with gynaecological neoplasms (i.e. vulva, ductal carcinoma of the breast) [2–4, 9, 11].

Paraneoplastic syndromes are often associated with ovarian cancers, as determined from a clinicopathological study of 908 patients in 1992 by Hudson and colleagues [12]. Few unspecific dermatological conditions were recorded. Only one patient was diagnosed with acanthosis nigricans maligna, the first (rare) dermatosis truly associated with malignant processes predominantly occurring in abdominal cancers (90 %), mostly consisting of adenocarcinomas. One patient showed dermatomyositis, which affects the skin and muscles. Clinical findings include malar erythema and poikiloderma on the thorax, associated with symmetric proximal paresis. Approximately 10–25 % of cases of dermatomyositis are paraneoplastic. Ovarian, pulmonary, gastric and genital carcinomas are most frequently correlated [13]. Hudson’s study showed other findings associated with ovarian carcinoma including haematological, neurological, endocrine and osteoarticular conditions.

In 2011, Fader et al. described the second case (the first reported in 1983) of Sweet’s syndrome in ovarian carcinoma [14]. Sweet’s syndrome or acute febrile neutrophilic dermatosis is characterized by painful, oedematous, shiny erythematous nodules or plaques, primarily on the face, neck, and extremities. Most neoplastic associations involve haematologic neoplasms [8, 13].

Dermatological signs and symptoms can be common paraneoplastic manifestations of underlying malignancy. Cutaneous lesions, typically associated with other neoplasms than ovarian carcinoma, are amongst the ones described above: Necrolytic migratory erythema (most commonly associated with islet cell tumours), Paraneoplastic Pemphigus (characterized by painful mucosal erosions, ulcerations and polymorphic skin lesions on the trunk and extremities, associated to lymphoproliferative disorders), Leser-Trélat sign (sudden onset of seborrheic keratosis associated with adenocarcinomas of the gastrointestinal tract and also lymphoproliferative abnormalities) and other, more rare dermatoses [13].

## Conclusions

To the best of our knowledge, no case of acrokeratosis paraneoplastica in association with ovarian cancer has been described before. This is a rare but important paraneoplastic skin finding to be recognized, as dermatological symptoms can precede the associated malignancy, unlike that of our patient.

As the histopathological findings are unspecific, the diagnosis can only be made on the basis of a combination of clinical and histological criteria and the regression of the cutaneous symptoms during and after therapy of the underlying carcinoma.

### Consent

Written informed consent was obtained from the patient for publication of this case report and any accompanying images. A copy of the written consent is available for review by the editor of this journal.
